# Cognitive-behavioural therapy role in the prevention of psychosis

**DOI:** 10.1192/j.eurpsy.2021.1333

**Published:** 2021-08-13

**Authors:** M. Pinho, D. Martins, S. Carvalho

**Affiliations:** Department Of Psychiatry, Hospital de Magalhães Lemos, Porto, Portugal

**Keywords:** cognitive-behavioural therapy, Ultra-High Risk, psychosis, prevention

## Abstract

**Introduction:**

About 30% of individuals in ultra-high risk (UHR) of psychosis develop overt psychosis within 3 years, and about 40% of those who don’t will keep experiencing ongoing attenuated psychotic symptoms and persistent functional disability. During this prodromal period, it’s possible to prevent the transition to a first-episode psychosis.

**Objectives:**

To conduct a short review of literature on the role of cognitive-behavioural therapy (CBT) in preventing psychosis in ultra-high risk patients.

**Methods:**

We performed a literature search on PUBMED, using the query: “Cognitive Behavioral Therapy” [Mesh] AND “psychosis” AND “prevention”. We focused on data from systematic reviews, clinical trials and meta-analysis published on last 5 years, either in English or Portuguese.

**Results:**

Some authors claim cognitive-behavioural therapy (CBT) as first-choice treatment in clients with ultra-high risk (UHR) for psychosis. CBT aims to normalize extraordinary experiences with education and to prevent delusional explanations. On a Japanese study, the total score of Positive and Negative Syndrome Scale (PANSS) significantly improved on post-intervention and follow-up assessments, with large effect sizes observed. Teaching families to apply CBT with their offspring may bolster therapeutic gains made in time-limited treatment. CBT showed an 83% probability of being more effective and less costly than routine care.

**Conclusions:**

Patients with UHR for psychosis can be treated successfully with CBT to postpone and prevent the transition to a first-episode psychosis. CBT for UHR has been included in the European guidelines and awaits dissemination and implementation in mental health services.

